# Silicon Dioxide Nanoparticles Affect the In Vitro Digestion of Sodium Caseinate but Not the Formation and Functionality of Bioactive Peptides

**DOI:** 10.1002/fsn3.70084

**Published:** 2025-03-06

**Authors:** Nazım Sergen Mısırlı, Fahriye Ceyda Dudak, Seda Yildirim‐Elikoglu

**Affiliations:** ^1^ Graduate School of Science and Engineering Hacettepe University Ankara Turkey; ^2^ Department of Food Engineering Hacettepe University Ankara Turkey

**Keywords:** bioactive peptides, in vitro digestion, nanoparticles, SiO_2_, sodium caseinate

## Abstract

Silicon dioxide (SiO_2_) nanoparticles (NPs) are among the most commonly utilized inorganic NPs in the food industry because of their ability to enhance the quality of a variety of foods. In the present study, the impact of SiO_2_ on the NaCN structure and digestibility was examined. The study also aimed to ascertain the bioactive peptide formation as affected by the interactions between SiO_2_ and NaCN. The CD spectrum signals in the 250–290‐nm region were altered in the presence of SiO_2_ at 14 mg/mL, indicating an alteration in the tertiary structure of NaCN. Additionally, the hydrodynamic size of NaCN micelles increased nearly 2‐fold upon interaction with SiO_2_. Gathering of casein micelles was observed in the presence of SiO_2_, especially at high NP concentrations. At the end of the in vitro digestion simulation, SiO_2_ led to a reduced proteolysis rate of NaCN from 8.3 mM Leu to 6.9 mM Leu in the stomach and from 17.4 mM Leu to 15.3 mM Leu in the intestine, as revealed by the OPA assay. The observed phenomenon is likely attributed to the aggregation of sodium caseinate following its interaction with SiO_2_ NPs, as evidenced by electron microscope imaging. However, there were no significant alterations in the overall peptide profile. Antioxidant, antimicrobial, and ACE‐inhibitory properties of the < 3‐kDa peptide fraction remained unaffected by SiO_2_, which is consistent with a similar peptide profile.

## Graphical abstract

This study aimed to investigate the effect of SiO_2_ nanoparticles on sodium caseinate structure and digestibility. SiO_2_ nanoparticles change the secondary and tertiary structures of NaCN. Also, SiO_2_ nanoparticles influence in vitro digestibility of NaCN. However, it does not affect the bioactivity of the < 3‐kDa peptide fraction.

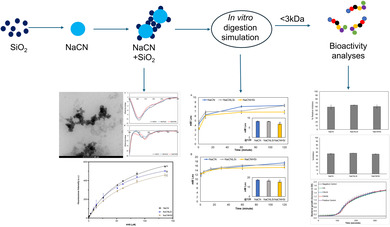

## 1 Introduction

The food sector has adopted substantial use of nanotechnology applications to increase product quality and shelf life in accordance with consumer demands. Among these applications, integrating various organic and inorganic nanomaterials into food can suggest potential improvements in final product characteristics. Silicon dioxide (SiO_2_), which is known as E551, is a food additive used as an anti‐caking and anti‐foaming agent in a variety of foods, including spices and dry powdered food mixes (Peters et al. 2012). The potential use of SiO_2_ in the dairy industry was mainly aimed at the modification of texture and flow characteristics of powdered dairy products (McClements and Xiao 2017).

Although SiO_2_ has been observed to exist in food additives as micro‐sized aggregates and agglomerates, it has been demonstrated that nano‐sized particles (< 100 nm) are also found in additive silica (Dekkers et al. 2011; Yang et al. 2016). In addition, it has been claimed that a variety of processing conditions and gastrointestinal (GI) system conditions during digestion might induce the aggregates to break down into nano‐sized particles (Dekkers et al. 2011; Fröhlich and Fröhlich 2016; Peters et al. 2012). Furthermore, the aggregation–dissociation behavior of inorganic NPs has also been reported to be affected by the presence of biomolecules in the medium (Basu et al. 2007; Givens et al. 2017; Sasidharan et al. 2015; Wang et al. 2020), which is critical for food system studies.

Because of their potential toxicity, there is an increasing concern about the detrimental impacts on human health of NPs even though they provide improved techno‐functional features. EFSA report in 2018 revealed no indication of SiO_2_ toxicity in humans at the reported use and levels. However, they also pointed out the lack of sufficient available toxicological data to estimate an acceptable daily intake (ADI) value (Additives et al. 2018). Recent research on the toxicity of SiO_2_ NPs revealed the harmful effects of these materials, which affect several systems in the body, including the digestive, respiratory, nervous, and circulatory systems (Deng et al. 2021; Feng et al. 2020; Ko et al. 2020; Wei et al. 2020; Yang et al. 2017). The importance of the presenting medium and its constituents on NP behavior and toxicity has been clearly shown by several researchers (Li, Jiang, et al. 2021; McClements et al. 2016; McClements and Xiao 2017; Zhang et al. 2019). It has been observed that the ‘corona’ layer that forms on the surface of NPs as a result of interactions with biomolecules alters the NPs' surface properties, which may change their reactivity, toxicity, and aggregation/dissociation behavior (Durán et al. 2015; Li, He, et al. 2021; Liu et al. 2020; Yallapu et al. 2015). Therefore, recent research on NPs has focused on simulated or real food matrix studies (Bae et al. 2018; Ersöz et al. 2022; Go et al. 2017; Laloux et al. 2020; McClements et al. 2017).

NP behavior and toxicity have been studied widely as affected by protein corona formation. However, little research was conducted on the consequences of these interactions on proteins. It was shown that the extent of protein hydrolysis during in vitro digestion simulation was decreased in the presence of inorganic NPs, which might lead to significant consequences (Cao et al. 2019). Recently, Martín‐Hernández et al. (2021) evaluated the effect of magnetic silica particles on the digestion of skimmed milk powder, peanuts, and tofu. They concluded that the in vitro digestion process was not affected by the presence of magnetic silica NPs. Peptide identification analyses revealed that especially long‐sequenced digestion‐resistant peptides were found in the protein corona and thought to be critical in the course of translocation and uptake of NPs in the intestine (Martín‐Hernández et al. 2021).

Beyond serving as nutrients, proteins also exhibit bioactive properties, with much of this activity ascribed to peptides inherent in their native sequences (Shahidi and Zhong 2008). It is well known that one of the important sources of bioactive peptides is milk proteins (Auestad and Layman 2021; Nielsen et al. 2023) Possible bioactivities of these peptides reported are antioxidant (Elias et al. 2006; Hernández‐Ledesma et al. 2007), anticancer (Azuma et al. 2000; Picot et al. 2006), antihypertensive (Chatterton et al. 2006; Hernández‐Ledesma et al. 2007; Maeno et al. 1996), antimicrobial (Benkerroum 2010; Pellegrini et al. 2001), anti‐inflammatory (Mukhopadhya et al. 2014), opioid, etc. (Shahidi and Zhong 2008). One of the most common ways of forming bioactive peptides is the enzymatic hydrolysis of proteins (Rizzello et al. 2016; Shahidi and Zhong 2008). Furthermore, the degree of hydrolysis of proteins has been reported to affect the functional properties of bioactive peptides formed from precursor proteins (Chen et al. 2012).

In the present study, the effects of the interactions between NaCN and SiO_2_ NPs were investigated in the course of protein structure and digestibility, with a specific emphasis on bioactive peptides. For this purpose, the possible alterations in the structure of NaCN were evaluated with different approaches. Then, samples were subjected to an in vitro digestion simulation, and the proteolysis rate of NaCN was evaluated. Finally, peptides with molecular weights less than 3 kDa were separated from the peptide mixture released in the final stage of digestion and investigated for their antioxidant, antimicrobial, and ACE‐inhibitory properties. We believe that our work provides valuable information and insights into the NP–protein interactions from a relatively less explored perspective.

## 2 Materials and Methods

### 2.1 Materials

Sodium dodecyl sulfate (SDS), ammonium persulfate (APS), acrylamide, bisacrylamide, TEMED, and Coomassie brilliant blue G‐250 were purchased from Bio‐Rad Laboratories (Watford, UK). All other chemicals were purchased from Sigma‐Aldrich Chemicals (St. Louis, MO, USA). Silicon dioxide NPs were purchased from Nanografi Nanotechnology A.S. (Ankara, Turkey) as a solution in deionized water (26%, w/w).

### 2.2 Characterization of SiO_2_ NPs

The morphology and properties of purchased SiO_2_ NPs were characterized via environmental scanning electron microscopy (ESEM), transmission electron microscopy (TEM), dynamic light scattering (DLS) measurements, and X‐ray diffraction (XRD) analyses.

For ESEM analyses, the SiO_2_ solution was dried on an aluminum stub and sputter‐coated with a 10‐nm gold layer. Then, the prepared samples were examined at an accelerating voltage of 15 kV with 100,000× magnification with an FEI‐Quanta 200 FEG (Thermo Fisher Scientific Co., Waltham MA, USA). To obtain TEM images of SiO_2_ NPs, the NP solution was dropped on the TEM grid; then, images were taken with an FEI Tecnai G2 Spirit BioRwin (Thermo Fisher Scientific, Massachusetts, USA).

The hydrodynamic diameter of SiO_2_ was determined via DLS measurements using Zetasizer Nano Zs (Malvern Panalytical Ltd. Malvern, UK). All measurements were conducted at 25°C. The refractive indices of the solvent (deionized water) and NPs were set at 1.34 and 1.45, respectively. The hydrodynamic diameter was calculated using the Stokes–Einstein equation.

The crystalline structure of SiO_2_ was determined by X‐ray diffraction measurements (PANalytical X'Pert3 Powder, Malvern Panalytical Ltd., U.K.). The liquid sample was dried to make the NP samples for analysis. XRD measurements with Cu‐Kα radiation (45 kV, 40 mA) were performed in the 10°–80° (2θ) (*λ* = 0.154 nm) range, with a scan step size of 0.026°.

### 2.3 Interaction of NaCN and SiO_2_

NaCN solution was prepared at a concentration of 3.4 g/100 mL in 50 mM phosphate buffer (pH 6.8) representing the average protein content in milk (Averdunk and Krogmeier 2011). SiO_2_ stock solution (26%, w/w) was sonicated in an ultrasound bath (VWR USC 200TH, VWR International, Malaysia) and then added to NaCN solution at a final concentration of 3.5 mg/mL (NaCNLSi) and 14 mg/mL (NaCNHSi). After the addition of SiO_2_ to NaCN, the mixture was vigorously shaken for 1 h at 25°C. The final concentrations of SiO_2_ in the samples were chosen based on the estimated daily intake of SiO_2_ NPs (3.5 mg/mL) (Dekkers et al. 2011) (NaCNLSi), and a higher concentration of the estimated daily intake was also examined (NaCNHSi). NaCN solution (%3.4, w/v) without NP addition was used as control, and all solutions were prepared in duplicate for further analysis.

#### 2.3.1 Circular Dichroism (CD)

CD spectroscopy measurements were conducted by using JASCO (J‐815 (Easton, PA, USA)) to determine structural alterations in NaCN upon interaction with SiO_2_. A quartz cuvette with a 1‐mm path length was used. CD spectra of incubated samples were obtained in the 190–250‐nm and 250–350‐nm ranges for far‐UV and near‐UV measurements, respectively. A scanning speed of 100 nm/min, a sensitivity of 100 mdeg, a bandwidth of 1 nm, and a response time of 4 s were set as instrumental parameters.

#### 2.3.2 Dynamic Light Scattering (DLS)

The hydrodynamic diameter of NaCN micelles was determined with the Zetasizer Nano Zs (Malvern Panalytical Ltd., Malvern, UK). After the incubation of SiO_2_ and CN at room temperature, samples were diluted with deionized water. All measurements were conducted at 25°C. The refractive index values of the solvent (PB) and NaCN were set at 1.34 and 1.39, respectively.

#### 2.3.3 Surface Hydrophobicity

The surface hydrophobicity of NaCN micelles was determined via fluorescence spectrophotometry according to Yildirim and Erdem (2015). 1‐ Anilinonaphthalene‐8 sulfonate (ANS) was used as the fluorescence probe. Briefly, NaCN in PB (50 mM, pH 6.8) was titrated with increasing concentrations of ANS (0–136 μM) and the fluorescence intensity resulting from the interaction of ANS with proteins was recorded. Fluorescence measurements were conducted at *λ*_ex_: 390 nm and *λ*_em_: 480 nm. The titration curves obtained from measurements were fitted with the Langmuir isotherm model by GraphPad (GraphPad Software, San Diego, California, USA). The measurements were conducted as 3 parallels.

#### 2.3.4 Electron Microscopy

ESEM and TEM images were obtained to investigate the possible changes in the colloidal structure of casein micelles after interaction with SiO_2_ NPs. For this purpose, samples were analyzed as described in section 2.2.

### 2.4 In Vitro Digestion

The harmonized model of 3‐stage in vitro digestion simulation was used according to (Minekus et al. 2014) with small modifications. The details of the digestion simulation are briefly summarized in Figure S1. Firstly, simulated saliva fluid (SSF), simulated gastric fluid (SGF), and simulated intestinal fluid (SIF) solutions were prepared. All digestion fluids were heated up to 37°C before use. NaCN solutions with and without SiO_2_ NPs were then mixed with SSF 1:1 by volume and incubated for 2 min at 37°C. Subsequently, oral bolus mixed with SGF (2000 U/mL pepsin, pH ≈ 2.0) 1:1 and incubated for 2 h at 37°C with gentle shaking. Later, gastric chyme was diluted with equal volumes of SIF (80 mM bile salt, 0.5 mg/mL 8XUSP pancreatin, pH ≈ 7.5) and incubated for 2 h at 37°C. A 50 mM phosphate buffer (PB, pH 6.8) without NaCN and NPs was used as the control for in vitro digestion. Samples were collected throughout the gastric and intestinal digestion at different time intervals (Gastric samples at first minute: G1; 10 min: G10; 30 min: G30; 60 min: G60; Intestinal samples at first minute: I1; 5 min: I5; 15 min: I15; 30 min: I30; 60 min: I60 and 120 min: I120). The pepsin and trypsin activities were inhibited by the immediate addition of 4 μL pepstatin A (1.82 mM) and 50 μL Pefabloc SC (0.1 M), respectively, for the gastric and intestinal samples taken at specific time intervals. Then, the samples were aliquoted and stored at −80°C for further analyses.

#### 2.4.1 Evaluation of Proteolysis During In Vitro Digestion

The degree of proteolysis during in vitro digestion was evaluated by the o‐phytaldehyde (OPA) method as described by Lorieau et al. (2018) and Bavaro et al. (2021) with small modifications. The oral stage was not analyzed in the course of protein hydrolysis since this study deals with protein hydrolysis.

OPA reagent (50 mL) was prepared with 20 mM sodium tetraborate (47.5 mL), 20% SDS (1.25 mL), β‐mercaptoethanol (25 μL), and 10‐mg/mL OPA (1.25 mL in ethanol) and stored in the dark for a maximum period of 24 h. Finally, 200‐μL OPA buffer and 10 μL digested samples were mixed in the 96‐well plate following incubation in the dark for 15 min. The UV absorbance of samples at 340 nm was measured with Synergy H1 Hybrid Multi‐Mode Microplate Reader (BioTek Instruments Inc., Vermont, USA). The amount of free amino groups released during in vitro digestion was calculated using L‐leucine as a standard.

SDS‐PAGE (Laemmli 1970) was carried out to investigate changes in the protein profile of the samples throughout digestion. For this purpose, samples taken at predetermined time intervals throughout digestion were mixed with SDS sample buffer and denatured at 95°C for 5 min. The proteins were separated with a resolving gel concentration of 17% by using a Mini‐PROTEAN Tetra Vertical electrophoresis chamber and Bio‐Rad PowerPac‐300 (Bio‐Rad, Watford, UK). Gels were stained with Coomassie brilliant blue G‐250 and were visualized using Agfa FotoLook software (Agfa‐Gevaert Group, Mortel, Belgium).

For peptide profile investigation, trichloroacetic acid (TCA) soluble fractions of the samples were analyzed via RP‐HPLC. TCA was added to the samples with a final concentration of 12%, and subsequently, the samples were centrifuged at 10000 *g* for 5 min. After centrifugation, the supernatant was filtered through a 0.45‐μm nylon filter before analysis. Peptide profiles of the samples were analyzed via Agilent 1100 series HPLC with a DAD detector. The separation was performed on an ACE C18 column (250×4.6 mm, 5 μm, 300 A°) which was kept at 30°C. Deionized water containing 0.1% Trifluoroacetic acid (TFA) was used as mobile phase A, and acetonitrile containing 0.1% TFA was used as mobile phase B. The elution was performed by isocratic elution with mobile phase B increasing by 40% for 100 min and monitored at 214 nm.

#### 2.4.2 Collection of < 3‐kDa Peptide Fraction

At the end of the intestinal digestion, the peptide fractions containing < 3‐kDa peptides were obtained by using Amicon Ultra 0.5‐mL centrifugal membranes with a molecular weight cutoff of 3 kDa (EMD Millipore; Billerica, MA, USA). The filtrates were lyophilized and stored at −20°C for further analyses. The freeze‐dried samples were resolved in pure water before being subjected to functionality experiments. The same procedure was carried out for the digestion mix without NaCN and SiO_2_, as a control.

#### 2.4.3 Bioactivity of < 3‐kDa Peptide Fraction

The antioxidant activity of < 3‐kDa peptide fraction was determined according to Re et al. (1999). For this purpose, ABTS^+^ (2,2′‐azino‐bis(3ethylbenzothiazoline‐6‐sulfonic acid)) was produced by reacting 7 mM ABTS stock solution and 2.45 mM potassium persulfate and keeping this mixture in the dark at room temperature for 12–16 h. Later, the solution was diluted with 50 mM PB (pH 7.4) to an absorbance of 0.7–0.8 at 734 nm after equilibration at room temperature. Subsequently, 10 μL samples were mixed with 1‐mL diluted ABTS^+^ solution and rested for 10 min at room temperature. ABTS^+^ scavenging was measured at 734 nm using a Synergy H1 Hybrid Multi‐Mode Microplate Reader (BioTek Instruments Inc., Vermont, USA). The results are expressed as “inhibition%,” which was determined using the following equation:

(1) $$\%\text{Inhibition}=\frac{A734\mathrm{nm}\;\text{control}-A734\mathrm{nm}\;\text{sample}}{A734\mathrm{nm}\;\text{control}}\times 100$$

Antimicrobial activity was determined by broth inhibition protocol according to Alvarez‐Ordóñez et al. (2013) with some modifications. *E. coli* K12 was chosen for investigation of antimicrobial activity. Firstly, the stock *E. coli* was incubated overnight in Luria‐Bertani (LB) broth. After incubation, it was subcultured into fresh LB (2% inoculum) and grown to the mid‐log phase (OD_600_ of 0.3–0.5). Then, bacteria cells were harvested by centrifugation at 2000 *g* for 10 min. The resulting pellets were washed once with phosphate‐buffered saline (PBS, pH 7.4) and resuspended in PBS. Following the addition of peptide samples and bacterial suspensions to new LB, OD_600_ values were monitored with Synergy H1 Hybrid Multi‐Mode Microplate Reader (BioTek Instruments Inc., Vermont, USA). Digestive fluids without peptides and PBS were chosen as positive and negative control samples, respectively. The growth ‘inhibition/activation %’ values were expressed in relation to the control samples after 60, 190, and 360 min. Inhibition/activation rates were calculated according to Equation (2) where OD_600_ control is the OD_600_ value for the control bacterial curve with addition of digestion media after indicated time, and OD_600_ sample is the OD for bacterial curve with the addition of the samples after indicated time

(2) $$\text{Inhibition}/\text{Activation}\%=\frac{\mathrm{OD}600\;\text{control}-\mathrm{OD}600\;\text{sample}}{\mathrm{OD}600\;\text{control}}\times 100$$

The ACE inhibition activity of < 3‐kDa peptides was monitored via an ACE inhibition kit (ACE1 Inhibitor Screening Kit (Colorimetric)‐MAK422, Sigma‐Aldrich). The kit utilizes the ability of an active ACE1 to hydrolyze a synthetic substrate, which results in a decrease in OD at 345 nm. Using the kit protocol, the percentage of inhibition, which represents the peptide mixture's inhibition potential, was determined.

### 2.5 Statistical Analysis

All data were presented as the mean and standard deviation (SD) from at least two independent experiments. The statistical significance was determined by one‐way analysis of variance (ANOVA) with the Tukey post hoc test unless otherwise indicated. *p* < 0.05 was considered statistically significant.

## 3 Results and Discussion

### 3.1 Characterization of SiO_2_ NPs

**FIGURE 1:**
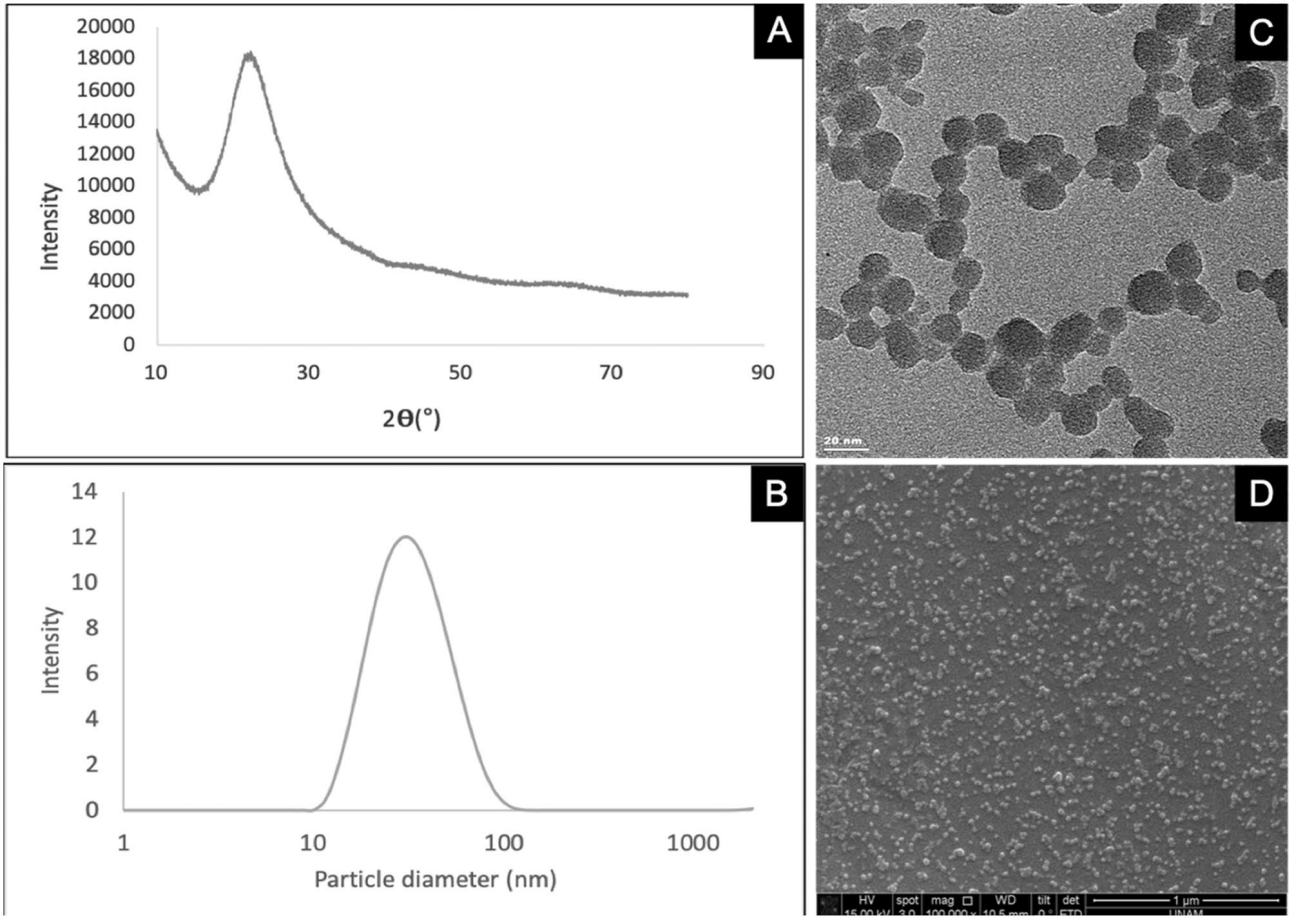
X‐ray diffraction pattern (A), particle size distribution (B), TEM (C), and ESEM (D) images of SiO_2_ NPs.

XRD was used to investigate the crystalline nature of SiO_2_ NPs. Figure 1A shows the typical characteristic X‐ray diffraction pattern of SiO_2_ with a broad peak observed at 21° corresponding to the amorphous crystalline phase (Dubey et al. 2015). The SiO_2_ NPs had an average particle diameter of 32.7 nm and showed a very low polydispersity index, as shown in Figure 1B. According to ESEM and TEM images, SiO_2_ NPs in solution were found to be highly dispersed or form small‐scale agglomerates rather than large‐scale clusters (Figure 1C,D).

### 3.2 Effect of SiO_2_ on NaCN Structure

**FIGURE 2:**
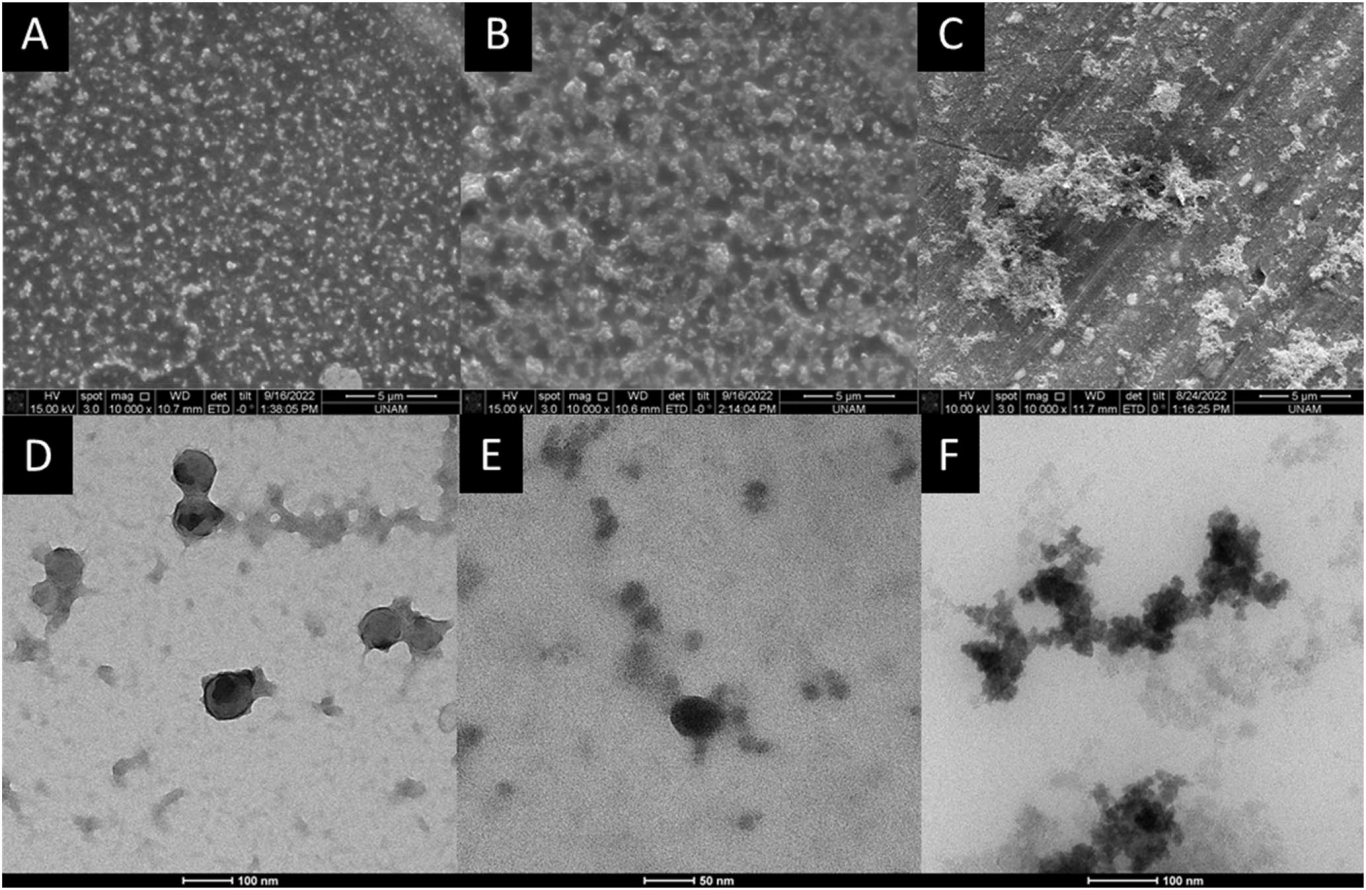
ESEM (A–C) and TEM (D–F) images of NaCN in the absence and presence of SiO_2_ nanoparticles (A, D: NaCN; B, E: NaCNLSi; C, F: NaCNHSi).

Morphological characteristics of NaCN upon interaction with SiO_2_ were investigated by electron microscopy. ESEM images of the samples revealed the gathering of NaCN micelles in the presence of SiO_2_, which reaches micron‐sized aggregates for NaCNHSi concentration (Figure 2A–C). TEM investigations were used to further assess the interactions between SiO_2_ and NaCN, and it appears that NaCN micelles were surrounded by SiO_2_ particles, which is more apparent for CNHSi (Figure 2B,E). We believe that the aggregation observed in ESEM images may have been induced by the steric destabilization of NaCN micelles caused by the interactions between NaCN and SiO_2_. For CNLSi, the SiO_2_ NPs could also be seen around the micelles, but probably because of the lower concentration compared to CNHSi, an obvious effect could not be observed. Cao et al. (2019) reported the loss of micelle shape‐like structure of caseins and the formation of nebulous aggregates upon interaction with relatively higher concentrations of TiO_2_ NPs (Cao et al. 2019). Similarly, in our case, larger aggregates are formed upon interaction with a high concentration of SiO_2_ (Figure 2C) but without any disruption of micelles (Figure 2F). Aggregation of proteins can occur concurrently with NP aggregation as a result of proteins adhering to NP surfaces. It has been reported that, in particular, when small peptides or globular proteins interact with NPs, they either randomly aggregate or self‐assemble via an ordered β‐sheet structure (Kim et al. 2016). In the case of casein micelles, due to the flexible structure and relatively higher micelle sizes, SiO_2_ NPs seem to form clusters on casein micelles, which might eventually act as a cross‐linking agent (Cao et al. 2019).

**FIGURE 3:**
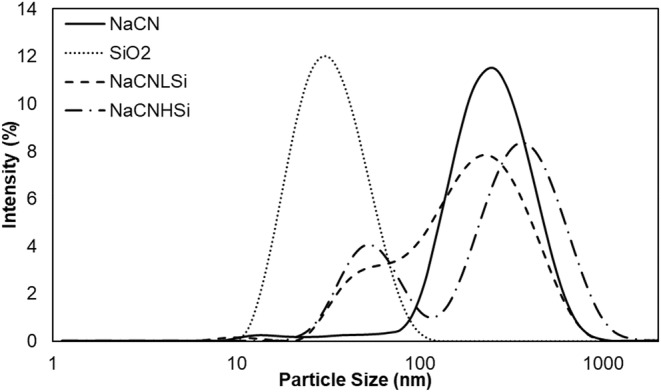
Dynamic light scattering spectrum of NaCN, SiO_2_ NPs, and NaCN after interaction with different concentrations (3.5 mg/mL (NaCNLSi) and 14 mg/mL(NaCNHSi)) of SiO_2_.

The hydrodynamic size distributions of NaCN, before and after incubation with SiO_2_, are displayed in Figure 3. The average size of NaCN and SiO_2_ was found to be around 200 and 30 nm, respectively. In technical terms, NaCN is obtained by precipitation of casein micelles at pH 4.6 and removing calcium phosphate by washing the precipitate followed by the addition of NaOH to resolubilize the caseins. It has been demonstrated that casein micelles in NaCN have smaller diameters than native micelles in milk and that their size is affected by environmental conditions such as ionic strength (Thomar and Nicolai 2015). The diameter of NaCN micelles was reported to be about 80 nm in the literature (HadjSadok et al. 2008; Thomar and Nicolai 2015), which is in accordance with our TEM images (Figure 2D). The possible explanation for the larger hydrodynamic diameter calculated by DLS measurements could be the aggregation of NaCN micelles, as suggested by Hemar et al. (2021), which was also supported by ESEM images of the present study (Figure 2A).

Upon interaction with SiO_2_, the micelle size distribution of NaCN increased from nearly 200 nm to 400 nm for NaNCHSi. This finding is in good agreement with the TEM images showing NaCN–SiO_2_ interactions (Figure 2F) which predictably lead to greater hydrodynamic radii, not surprisingly. However, due to the lack of the ability of DLS analysis to distinguish between micron‐sized particles in a polydisperse medium, the large‐scale aggregation shown by ESEM could not be ascertained via light scattering measurements (Caputo et al. 2021; Wohlleben 2012). On the other hand, the main casein peak was widened by the interaction with SiO_2_ at low concentrations and formed a shoulder at smaller diameters (about ≈80 nm), as seen in Figure 3, which depicts the high polydispersity index observed in NaCNLSi. This finding is supported by TEM and SEM images of NaCNLSi (Figure 2B,E) showing the variously sized clusters in the sample. Cao et al. (2019) reported that electrostatic interactions between inorganic titanium dioxide NPs and casein micelles cause an increase in the hydrodynamic diameter of casein micelles up to a certain NP: casein ratio. However, at higher NP concentrations, the micelles' structure was disrupted, resulting in a lower micelle size. In the present work, the hydrodynamic diameter of NaCN seems to increase after interaction with SiO_2_, indicating NaCN micelle aggregation, especially at high concentrations of SiO_2_.

**FIGURE 4:**
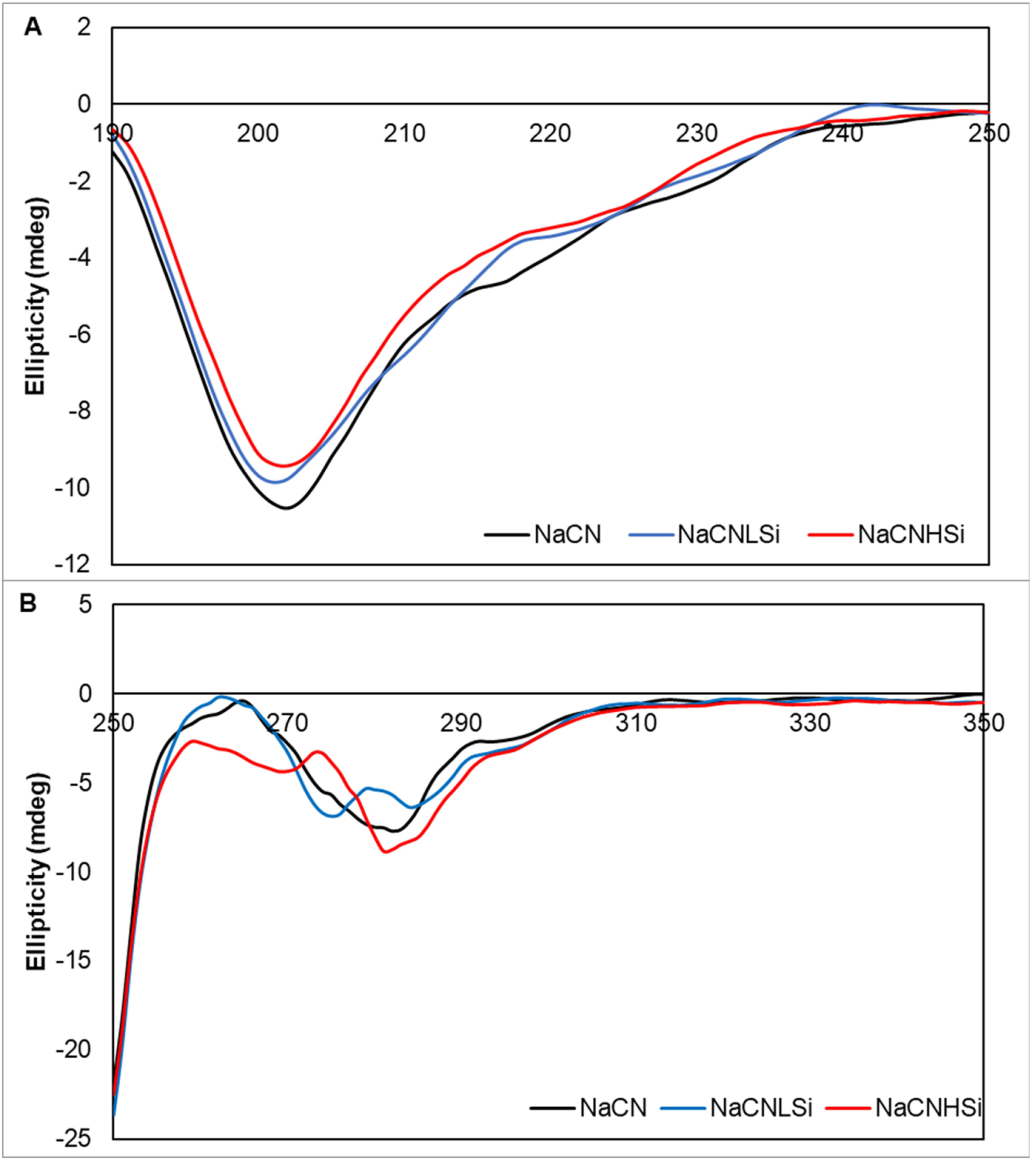
Far‐UV (A) and near‐UV (B) CD spectra of NaCN in the absence and presence of SiO_2_ (black: NaCN without SiO_2_ addition, blue: NaCN after interaction with 3.5 mg/mL of SiO_2_, red: NaCN after interaction with 14 mg/mL of SiO_2_).

The consequences of the interactions between SiO_2_ NPs and NaCN were further investigated in terms of conformational changes and surface hydrophobicity of NaCN micelles. CD spectroscopy is considered a useful and widely preferred tool to assess the conformational changes in protein structure by identifying the main structural elements. Figure 4 shows the CD spectrum (A: far‐UV; B: near‐UV) of NaCN with and without SiO_2_ addition. NaCN spectra showed one distinct negative band at ≈200 nm and a little shoulder between 210 and 230 nm, which corresponds to the randomly structured elements, and similar results were reported previously by Sun et al. (2018) and Zhan et al. (2018). Another characteristic positive peak at 215 nm for random coil was not detected for NaCN in accordance with the findings of Barreto et al. (2003) and Moreira et al. (2019). A less negative signal at 195–225 nm and a small red shift were observed for NaCNHSi, indicating a structural modification with a reduction in random structure and a promotion of ordered structure upon interaction with high concentration SiO_2_ (Chakraborty and Basak 2008). Similarly, Vitali et al. (2018) reported that interaction with α‐casein and SiO_2_ NPs led to a change in the secondary structure of α‐casein via a red shift of peak at 200 nm. On the other hand, the circular dichroism signal of NaCNLSi was not significantly different from that of NaCN.

Numerous studies have documented the impact of nanoparticles on the structural characteristics of proteins regarding protein coronas because it has been revealed that changes in protein structure that may occur during adsorption onto NP surfaces are essential to the functionality of NPs (Park 2020).

Generally, interaction with NPs leads to a transition from ordered structure to random coil, indicating partial unfolding or aggregation of proteins when they are adsorbed to the NP surface. It has been observed that silica NPs unfold the hen egg white lysozyme, forming fiber‐like protein aggregates. They reported a decrease in the protein's ordered structure while random structural elements improved when interacting with silica NPs (Konar et al. 2019). Wangoo et al. (2008) also demonstrated that the interaction between gold NPs and bovine serum albumin (BSA) influenced the secondary structure of BSA. They reported the conversion of α‐helical structure to a random structure in a concentration‐dependent manner. von Bergen et al. (2005) demonstrated that interaction with carbon nanotubes led to the aggregation of tau protein, which was driven by a transition from random coil to β‐sheet structure, similar to our findings in the present study revealing a reduction in random structural elements in NaCN when interacted with high‐concentration SiO_2_. Also, Vitali et al. (2018) concluded that while globular proteins tend to lose their ordered structure, the secondary structure content in intrinsically disordered proteins generally increases upon interaction with NPs, which is in good agreement with our finding since caseins are known to be the randomly structured proteins.

The effect of NPs on protein structure is considered to be affected by the molecular interactions affected by the type and surface properties of NP, as well as the structure of the protein. For example, (Ge et al. 2015) reported a well‐ordered and thermodynamically stable structure of bovine fibrinogen upon interaction with carbon nanotubes, while the interaction of γ‐Fe_2_O_3_ NPs led to a reduction in α‐helix and β‐sheet content of the same protein (Zhang et al. 2015). However, due to the micellar structure of NaCN, we observed the surroundings of NaCN micelles by SiO_2_ nanoparticles, differently from the abovementioned studies that primarily deal with protein corona structure.

The near‐UV spectra of the samples were also affected by the presence of SiO_2_ NPs (Figure 4B) with a more pronounced change observed for NaCNHSi. In the near‐UV CD spectrum, signals in the 250–270‐nm, 270–290‐nm, and 280–300‐nm regions are attributed to phenylalanine, tyrosine, and tryptophan, respectively (Rahman et al. 2013). The near‐UV spectrum profile of NaCN showed a negative broad band at ≈280 nm with a shoulder at ≈290 nm, and a positive peak at 265 nm, which is attributed to tyrosine and phenylalanine, respectively (Sun et al. 2018). The intensity of 270–290 nm centered at 280 nm decreased in the NaCNLSi spectrum. In comparison to NaCN, the negative peak intensity at 280 nm increased while the positive peak intensity at 265 nm decreased for NaCNHSi. Furthermore, a minimal red shift was noted for NaCNHSi. These changes indicate possible alterations in the tertiary structure of native NaCN via phenylalanine and tyrosine residues upon interaction with SiO_2_.

Overall, previous research has reported the structural changes that occur in several proteins when they interact with NPs (Jafari Azad et al. 2017; Zolghadri et al. 2009), although Bardhan et al. (2009) argued that the impact of protein–NP interactions on protein structural features was negligible. According to our findings, the CD spectrum assessment revealed that the secondary and tertiary structures of NaCN were affected by SiO_2_.

Our findings have been supported by surface hydrophobicity analyses. For this purpose, the samples were titrated with different concentrations of ANS (0–136 μM) and the fluorescence intensity resulting from the interaction of ANS with proteins was measured. ANS has been reported to interact with proteins mainly via hydrophobic attractions at non‐alkaline pH (Hawe et al. 2008). Thus, ANS titration curves provide information regarding the protein surface hydrophobicity and protein‐solvent interactions in solution. The maximum fluorescence intensity observed in ANS titration curves represents the maximum number of surface hydrophobic sites of casein micelles (Yildirim and Erdem 2015).

**FIGURE 5:**
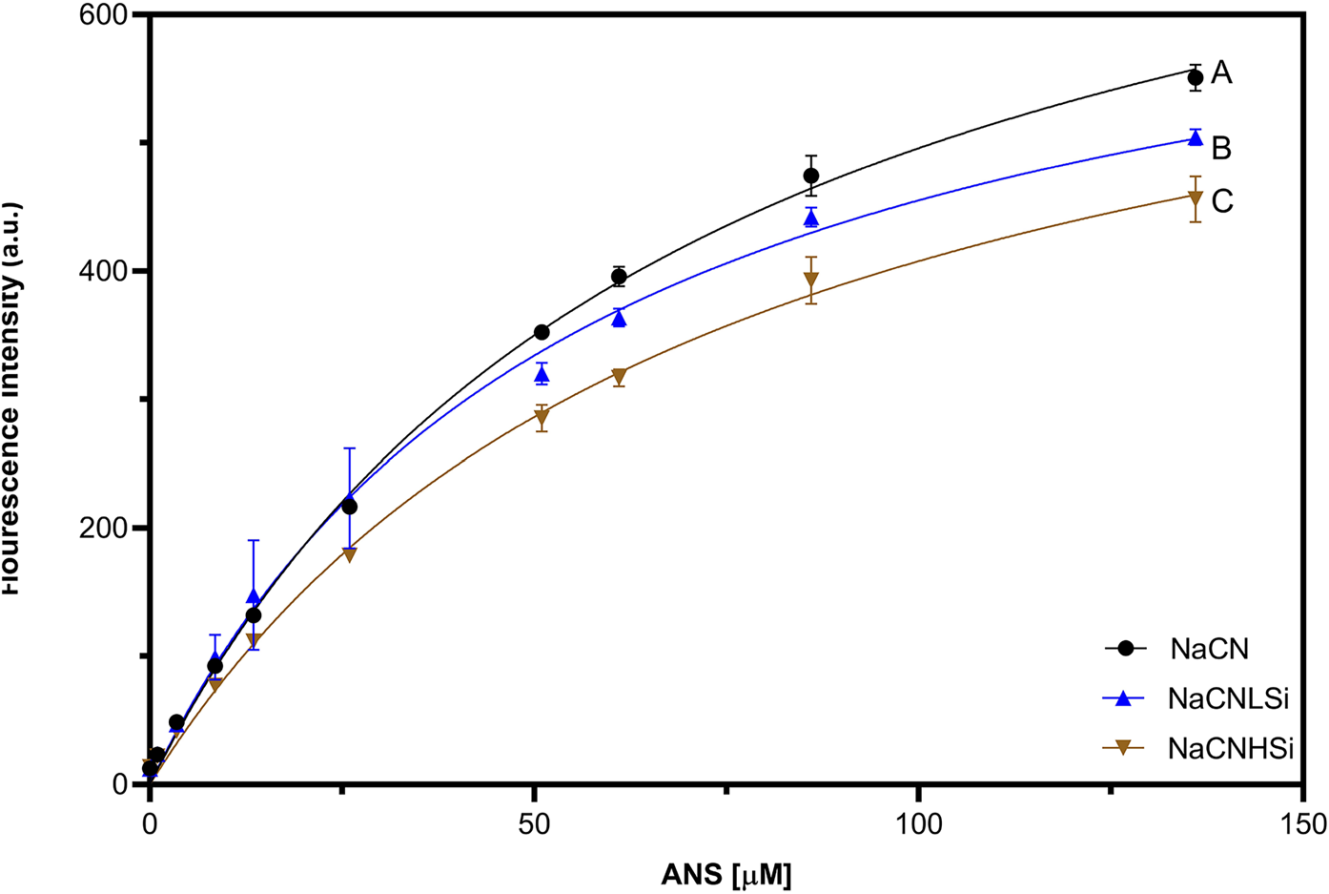
ANS titration curves of NaCN in the absence (black) and presence (blue and brown) of SiO_2_. Different letters indicate significant differences in fluorescence intensity at the end point of titration (*p* < 0.05). B_max_ obtained from fitting: NaCN: 850.9; NaCNLSi: 713.6 and NaCNHSi: 708.6.

The maximum fluorescence intensity at the highest ANS concentration was significantly decreased in a SiO_2_ concentration‐dependent manner (Figure 5). The corresponding decrease in surface hydrophobic sites of NaCN might be related to the alterations in casein structure upon interaction with SiO_2_, as previously discussed in this section. Another reason could be the potential hydrophobic interactions between NaCN and SiO_2_ because of their hydrophobic character (He et al. 2004; Puddu and Perry 2012), which might lead to the masking of surface hydrophobic sites of caseins and prevent ANS binding. As shown in CD analysis, the signals attributed to the hydrophobic amino acid residues such as phenylalanine changed after interaction with SiO_2_ NPs (Figure 4). Overall, CD and ANS findings indicate hydrophobic attractions between SiO_2_ NPs and NaCN followed by a decrease in surface hydrophobicity. However, further research on the characteristics of the interactions between NaCN and SiO_2_ is considered necessary for a better understanding.

### 3.3 Effect of SiO_2_ on the Hydrolysis of NaCN During In Vitro Digestion

**FIGURE 6:**
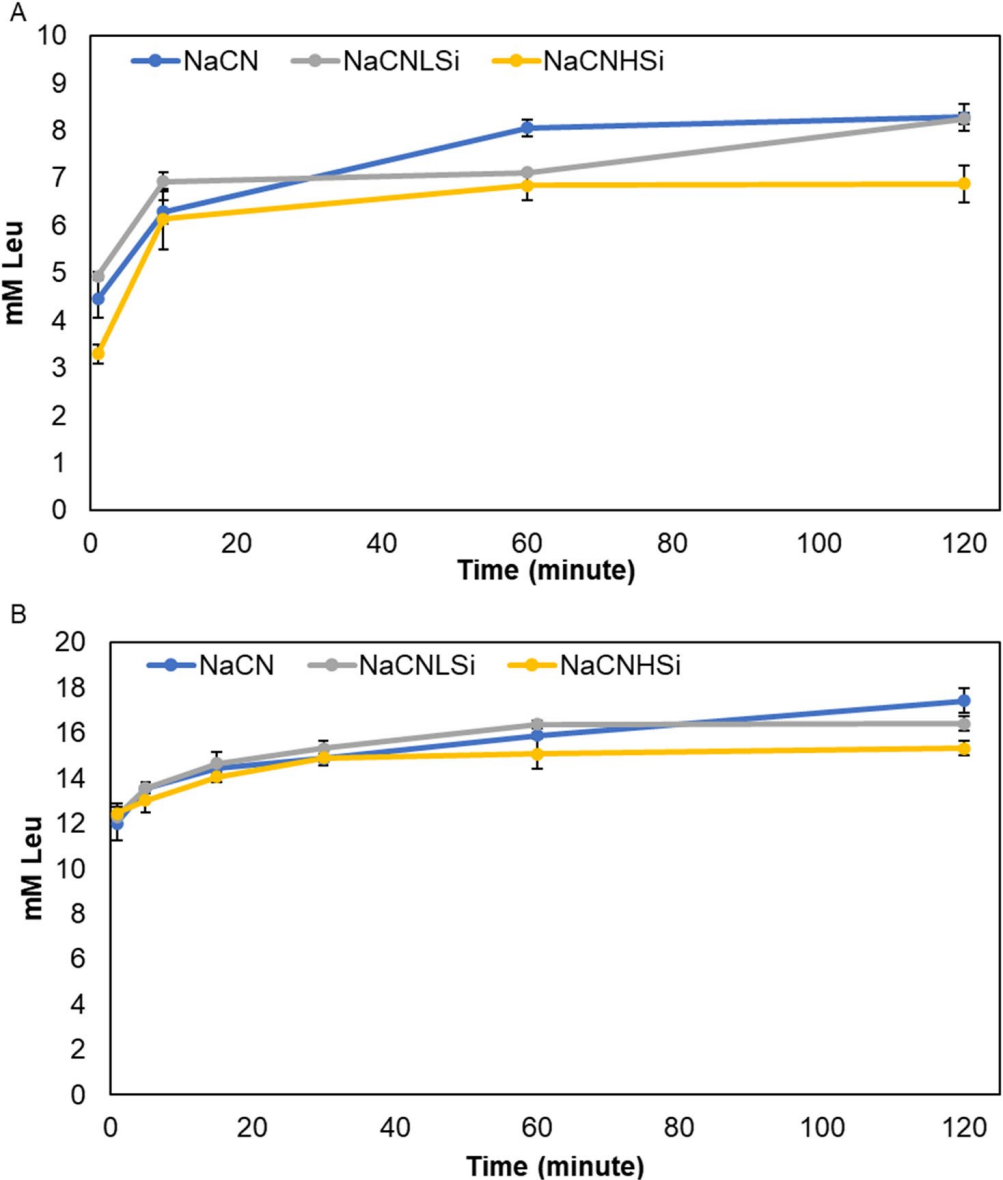
Hydrolysis degree of NaCN (blue), NaCNLSi (gray), and NaCNHSi (yellow) during gastric (A) and intestinal (B) digestion periods. Significant difference from NaCN (*p < 0.05; **p < 0.01) at the end of (120 min) each stage.

The impact of the SiO_2_ nanoparticles on the in vitro digestion of NaCN and protein hydrolysis was evaluated in the gastric and intestinal digestion periods. The oral phase was omitted due to the lack of proteolysis during oral digestion (Tagliazucchi et al. 2016). Figure 6 shows the amount of free amino groups released from NaCN over the gastric (Figure 6A) and intestinal (Figure 6B) digestion periods. It was determined that the hydrolysis degree was reduced significantly in the NaCNHSi at the final point of gastric digestion, whereas no significant difference could be observed for NaCNLSi compared to that of NaCN. It should be noted that at the very beginning of digestion, the degree of hydrolysis for NaCNHSi was also significantly lower than in other samples. The proteolysis degree of caseins in the intestine was very similar at the beginning for all samples. However, compared to the control, NaCNHSi and NaCNLSi had fewer amounts of free amino groups indicating a lower degree of hydrolysis at the end of intestinal digestion (Figure 6B).

Our findings revealed that the gastric and intestinal digestion was affected by the presence of SiO_2_ in a similar way, suggesting a lower degree of proteolysis of caseins, which was more apparent for NaCNHSi. Similarly, several studies have shown that the interactions between NPs and proteins resulted in a reduction in protein digestibility (Cao et al. 2019) (Di Silvio et al. 2016). However, a recent study performed with magnetic SiO_2_ NPs concluded the insignificant effect of NPs on protein hydrolysis during in vitro digestion (Martín‐Hernández et al. 2021). In the present study, the reduced colloidal stability and aggregation behavior of caseins upon interaction with SiO_2_ (Figure 2) might be the cause of the reduced digestibility. Because, as reported by Cao et al. (2019), modifications in the peptide bonds exposed to digestive enzymes limit proteolytic enzymes' ability to access the cleavage sites in caseins. Another possible explanation for the decreased casein hydrolysis level could be the reduction in the activity of proteolytic enzymes upon interaction with NPs in the medium, as claimed by many researchers; however, further research on the evaluation of molecular interactions is needed to make a more precise interpretation.

To evaluate the protein/peptide profile of the samples through each digestion step, SDS‐PAGE and RP‐HPLC analyses were performed. Figure S2 displays the electrophoretograms of the samples taken throughout gastric and intestinal digestion. Casein bands are more clearly apparent in the first minute of gastric digestion in the samples containing SiO_2_ NPs compared to SiO_2_‐free NaCN (Figure S2A) and yet the bandwidths were even denser for NaCNHSi. At the end of gastric digestion, none of the main casein bands were observed in the gels, indicating a complete proteolysis of intact proteins in the stomach in accordance with the findings of previous studies (Lorieau et al. 2018; Sheng et al. 2021; Tunick et al. 2016). The bands seen below 20 kDa at the first minute of digestion (Figure S2, lines 1, 5, and 9) are considered to be the long‐chain polypeptides released from intact proteins. The bandwidth of these fractions correlates negatively with the bandwidth of intact casein, as might be expected. These fractions seem to be degraded to smaller peptides at the further stages and led to the formation of a broad band at the bottom of the gels. According to PAGE analyses, NaCN seems to be hydrolyzed faster compared to NaCNLSi and NaCNHSi, which is in good agreement with our findings obtained by OPA analysis (Figure 6A). In addition, no significant change in the protein profile was detected at the end of gastric digestion as affected by SiO_2_. We consider that the alterations in NaCN micelle stability might cause the decreased proteolytic activity of digestive enzymes at the very beginning of gastric digestion. However, no significant change was observed for further proteolysis in the stomach. The lower bandwidth of peptides near the bottom of the gels at the first minute of intestinal digestion (Figure S2B, lines 1, 7, and 13) compared to the end of gastric digestion (Figure S2A, lines 4, 8, and 12) indicates that the peptides generated at the end of gastric digestion appear to have further degraded to extremely small peptides in the intestine. Additionally, these bands disappeared after 5 min of intestinal digestion, revealing the complete hydrolysis of peptides that are detectable at these SDS‐PAGE conditions. Contrary to gastric digestion, no significant effect of SiO_2_ NPs on NaCN digestion during the intestinal phase was observed. The reduced amount of free amino groups released at the end of intestinal digestion for SiO_2_‐added samples (Figure 6B) might probably arise from the differences in further degradation patterns that could not be monitored by SDS‐PAGE. It should also be noted that the behavior of NPs in the stomach and intestinal tract might be affected by environmental conditions such as pH, ionic strength, and biomolecules (Pinďáková et al. 2017; Walczak et al. 2013; Zhou et al. 2021) which could also affect the digestibility of proteins (Ersöz et al. 2022; Sun et al. 2020). However, these aspects are beyond the scope of the current study.

As revealed by CD spectroscopy findings (Figure 4), the interaction of NaCN with low‐concentration SiO_2_ affected the tyrosine residues while both tyrosine and phenylalanine residues were affected by the presence of SiO_2_ at high concentrations. Hence, the interactions between NaCN and SiO_2_ might alter the resistance of the residues mentioned above against pepsin activity in the stomach for NaCNHSi (Smith and Morton 2010).

At the end of the intestinal digestion, the hydrolysis of NaCN decreased in a concentration‐dependent manner with SiO_2_. Puddu and Perry (2012) demonstrated that, at neutral pH, lysine‐containing peptides interact with SiO_2_ NPs via different forces. Furthermore, Guo and Holland (2014) showed that lysine residues interact with SiO_2_ NPs, which alters the monolayer around SiO_2_ NPs at high salt concentration. In our case, SiO2 NPs probably interacted with lysine residues to reduce trypsin activity toward its substrate. Opposite to our findings, Martín‐Hernández et al. (2021) revealed no change in the hydrolysis level and peptide profile of several food matrices, including milk powder, during in vitro digestion, when magnetic SiO_2_ was added to samples. The size of the SiO_2_ NPs and the different protein sources, as well as the physical state of the medium (milk powder) that was used, could be the cause of this contradiction (Piella et al. 2017; Suvarna et al. 2018; Yadav et al. 2016).

TCA soluble fractions of the digesta at certain time intervals were analyzed via RP‐HPLC to further evaluate the peptide profile of the samples in the absence and presence of SiO_2_ NPs. The relevant chromatograms are shown in Figures S3 and S4. For gastric digestion samples, the peaks eluted between 70 and 90 min are considered to be formed by casein degradation at the first minute of gastric digestion. A lower peak area was calculated for NaCNHSi compared to that for NaCN and NaCNLSi, indicating a lower amount of peptides released when NaCN is incubated with a high concentration of SiO_2_. The peak area of the initially observed peaks between 70 and 90 min started to decrease from the 60th min of digestion, probably due to degradation to smaller peptides. Additional peaks eluted between 40 and 70 min observed for 30th, 60th, and 120th min of gastric digestion were the indicators of proceeding proteolysis. Similar results were reported for β‐casein (Schmelzer et al. 2007) and casein glycomacropeptide (Martinez et al. 2016) in the literature. The effect of SiO_2_ NPs on the eluted peak area was found to be insignificant (*p* > 0.05) except for the first minute of gastric digestion. These results are in good accordance with our SDS‐PAGE findings, while OPA results revealed a lower degree of proteolysis for the NaCNHSi at the end of gastric and intestinal digestion. This variation could be the result of different sample preparation procedures, wherein TCA precipitation was not used for the OPA assay and only TCA soluble peptides were examined by RP‐HPLC. Nearly none of the peptides produced in the stomach at the beginning of digestion are eluted during intestinal digestion, indicating that these peptides are rapidly broken down by strong trypsin activity, which supports the results of the SDS‐PAGE. Additionally, it has been established that the digestive fluid itself is the source of the dominating peaks found in the samples taken during intestinal digestion (data not shown). Furthermore, no discernible impact of SiO_2_ was seen on the peptide profile of the samples during intestinal digestion, supporting the earlier results in the present research.

Overall, SiO_2_ NPs did not affect the peptide profile during the in vitro digestion simulation. However, we demonstrated that SiO_2_ NPs reduced the proteolysis rate of NaCN during digestion. Similar to our findings, Cao et al. (2019) revealed that TiO_2_ NPs reduced the degree of casein hydrolysis in the stomach while not affecting intestinal digestion. The two enzymes that are mainly responsible for the proteolysis in the stomach and intestine, pepsin and trypsin, hydrolyze the proteins in different ways. Typically, pepsin cleaves the proteins after phenylalanine, leucine (Ahn et al. 2013), and aromatic amino acids (Cao et al. 2019) whereas trypsin mainly attracts the peptide bonds at the C‐terminal with lysine and arginine residues (Manea et al. 2007). This distinction could be responsible for the observed changes in the impact of SiO_2_ NPs on gastric and intestinal digestion processes along with different environmental conditions (Cao et al. 2019).

### 3.4 Bio‐Functional Properties of < 3‐kDa Peptide Fraction After In Vitro Digestion Simulation

A < 3‐kDa peptide fraction that was obtained via centrifugal filtration at the end of in vitro digestion was assessed to see the possible effects of SiO_2_ on the functional properties of the bioactive peptides. Antioxidant activity, antimicrobial activity, and ACE inhibition assays were carried out to examine the bioactivity of the < 3‐kDa peptide fraction.

**FIGURE 7:**
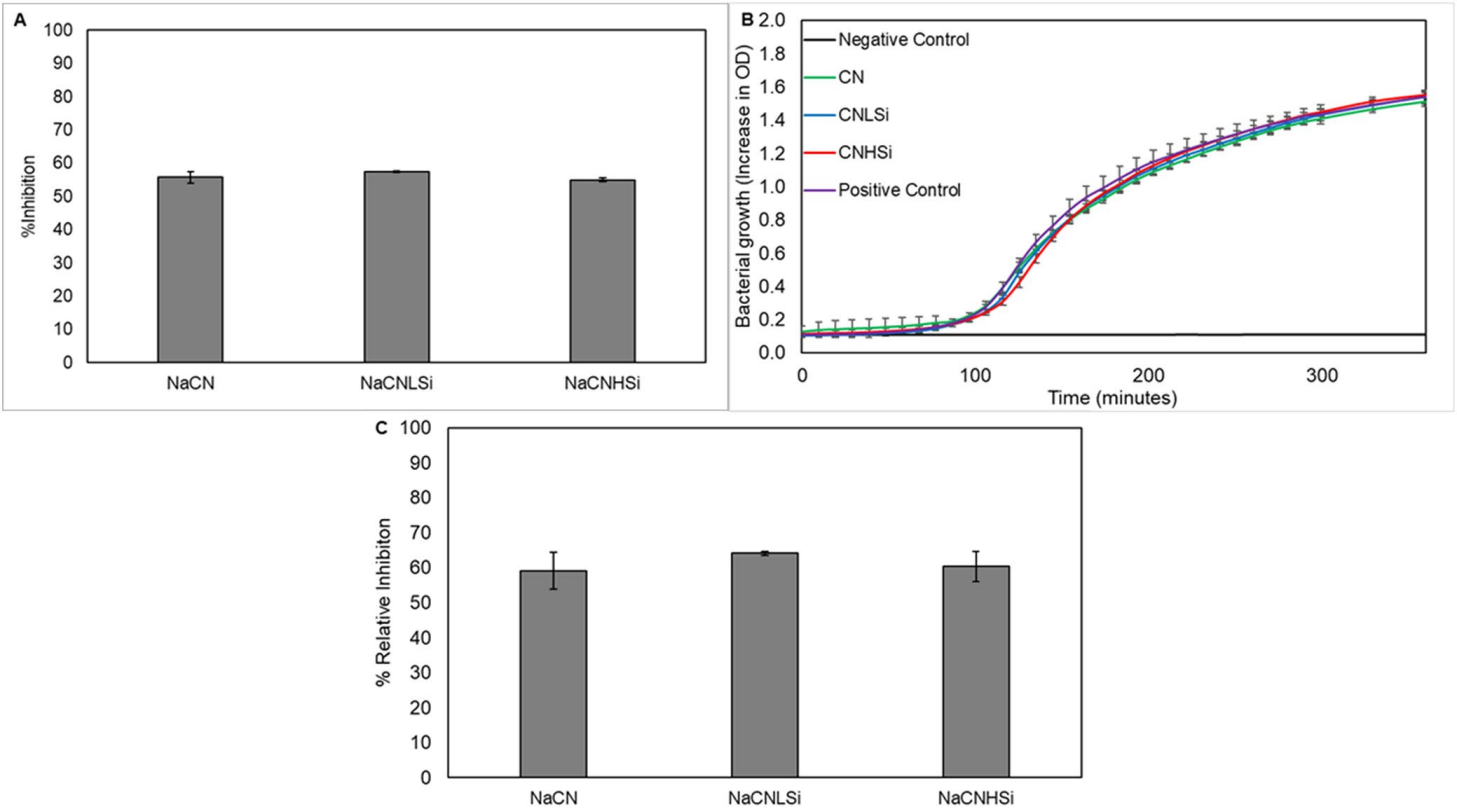
Antioxidant activity (A), bacterial growth of *E. coli* K12 (B), and ACE inhibition (relative inhibition %) of < 3‐kDA peptide fractions formed at the end of NaCN digestion in the absence and presence of SiO_2_.

ABTS analyses were performed to determine the potential antioxidant activity, and the results are shown in Figure 7A. A significant amount of antioxidant activity was observed for all samples in accordance with recent research that demonstrated the promising antioxidant capacity of milk protein hydrolysate (Bielecka et al. 2022). The effect of SiO_2_ on the formation of antioxidant peptides at the end of the digestion was found to be insignificant regardless of the NP concentration (*p* > 0.05).

To determine the antimicrobial activity of the peptide fraction, a broth inhibition assay was used. The inhibition of the peptide fractions on *E. coli* K12 was determined by measuring the optical density at 600 nm at different time intervals after incubation of medium with < 3‐kDa peptide fraction (Figure 7B). Table S1 shows the inhibition % values of the samples compared to positive control at 60, 190, and 360 min, which represent the lag phase, logarithmic phase, and stationary phase in the growth curve, respectively (Figure 7B). The effect of SiO_2_ on the antimicrobial activity of the < 3‐kDa peptide fraction was found to be insignificant, as revealed by representative inhibition % values in Table S1. In addition, none of the samples showed antimicrobial activity against *E. coli*. Although the antimicrobial activity of casein hydrolysates has been previously reported by several researchers (Benkerroum 2010; Bielecka et al. 2022), the relevant peptides were reported to be obtained by non‐tryptic digestion or larger than 3 kDa. Besides, peptides having a molecular weight smaller than 3 kDa could not be obtained after tryptic digestion, which could explain the inconsistent results (Benkerroum 2010).

ACE inhibition of < 3‐kDa peptide fraction was analyzed by ACE‐1 inhibition kit (MAK422, Sigma‐Aldrich). Figure 7C shows the “relative inhibition %” of the peptide fractions in the absence and presence of SiO_2_ NPs. All samples showed significant ACE inhibition levels compared to the control that contains enzyme and buffer solely. Accordingly, there was no significant difference in the ACE‐inhibitory activity of the peptide mixtures as affected by SiO_2_ (*p* > 0.05), similar to other biological functions.

In summary, though antioxidant and ACE‐inhibitory properties were observed in all samples, SiO_2_ NPs did not cause any significant change in these functional properties. Several studies in the literature reported the ACE‐inhibitory (Hong et al. 2015; Miguel et al. 2009; Tu et al. 2018) and antioxidant activities (Bielecka et al. 2022; Pihlanto 2006) of casein hydrolysates, in accordance with our findings. Peptides obtained in the present study likely exhibit features analogous to those documented in the literature, including antioxidant, ACE‐inhibiting, and antibacterial effects (Scudino et al. 2024; Kashung and Karuthapandian 2025). Although we demonstrated that SiO_2_ affected the hydrolysis degree during in vitro digestion simulation, we did not observe any differences in peptide fractions at the end of digestion in the absence and presence of SiO_2_ NPs (Figures S3 and S4). The reason for the unchanged bioactivity of the peptide fraction could be attributed to the identical peptide profiles at the end of the digestion simulation. Based on our findings, none of the peptide fractions that were obtained from the samples showed any antibacterial activity. However, we claim that the possibility of a change in the bio‐functional properties of the < 3‐kDa peptide fraction upon adsorption onto the surface of the NPs present in the environment after digestion cannot be evaluated within the scope of this study, because SiO_2_ NPs could not pass through the membranes having a 3‐kDa cutoff value used in the experimental procedure. It is expected that in the future, it may be advantageous to discover the functional properties of the peptides that might be presented in the corona layer during co‐digestion of SiO_2_ NPs and NaCN.

## 4 Conclusion

In the present study, we aimed to investigate the effects of the interactions between NaCN and SiO_2_ NPs on the structure and digestibility of NaCN, as well as the bioactivity of the peptide mixture formed upon digestion. Findings from this study revealed that upon interaction with SiO_2_, the secondary and tertiary structure of NaCN was altered. Also, hydrophobic interactions play a significant role in the binding of SiO_2_ NPs to NaCN micelles. Throughout digestion simulation, SiO_2_ NPs led to reduced proteolytic activity of digestive enzymes regarding NaCN hydrolysis, being more apparent for the concentration above the estimated daily intake amount of SiO_2_. Importantly, despite the alterations during digestion, there was no discernible influence of SiO_2_ NPs on the bioactive peptide formation. More detailed results could potentially be obtained through the identification of the bioactive peptides by means of mass spectrometry and bioinformatics. Nevertheless, we found no appreciable alterations in the profile and functionality of the < 3‐kDa peptide fractions. These findings hold significant ramifications for the food industry, as SiO_2_ nanoparticles are frequently utilized as additives in diverse compositions. It is essential to ensure that such NPs do not undermine the nutritional and functional attributes of protein‐based diets to preserve their intended health advantages. In view of the increasing demand for functional and fortified foods, our research emphasizes the applicability of SiO_2_ nanoparticles in the food sector, while underscoring the necessity for ongoing evaluation of their interactions with dietary proteins. Although the present study revealed that bioactive peptide formation was not affected by SiO_2_, future studies on the interactions between bioactive peptides and SiO_2_ NPs when present in the same medium, as well as the impact of these interactions on the bioavailability and cellular absorption of bioactive peptides, to ensure long‐term safety, are considered essential.

## Author Contributions

**Nazım Sergen Mısırlı:** formal analysis (lead), investigation (equal), methodology (lead), visualization (lead), writing – original draft (lead). **Fahriye** **Ceyda Dudak:** conceptualization (equal), resources (equal), supervision (equal), writing – review and editing (equal). **Seda Yildirim‐Elikoglu:** conceptualization (lead), funding acquisition (lead), project administration (lead), resources (lead), supervision (equal), writing – review and editing (lead).

## Ethics Statement

The authors have nothing to report.

## Conflicts of Interest

The authors declare no conflicts of interest.

## Supporting information


Appendix S1.

## Data Availability

The data that support the findings for this study are available on request from the corresponding author upon reasonable request.
