# Supplementary material for: Silicon Dioxide Nanoparticles Affect the In Vitro Digestion of Sodium Caseinate but Not the Formation and Functionality of Bioactive Peptides
Source: Food Sci Nutr. 2025 Mar 6;13(3):e70084. doi: 10.1002/fsn3.70084 (PMC11885160; doi:10.1002/fsn3.70084)
Supplement: Supplementary file 1 — Appendix S1. [file FSN3-13-e70084-s001.docx]

**SUPPLEMENTARY MATERIAL**

**Silicon dioxide nanoparticles affect the *in vitro* digestion of sodium caseinate but not the formation and functionality of bioactive peptides**

**Running title: Interaction of SiO2 particles with caseinate**

Nazım Sergen Mısırlı^1^, Ceyda Dudak Seker^2^ Seda Yildirim-Elikoğlu^2^*

^1^Graduate School of Science and Engineering, Hacettepe University, Beytepe, 06800 Ankara, Turkey.

^2^Department of Food Engineering, Hacettepe University, Beytepe 06800, Ankara, Turkey.

*corresponding author: Seda Yildirim-Elikoğlu

e-mail address: yildirims@hacettepe.edu.tr


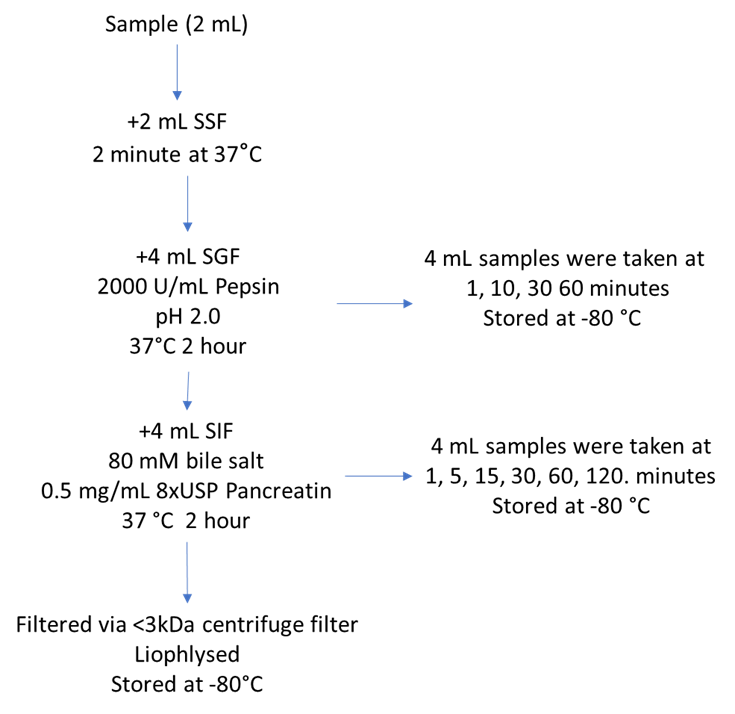


Figure S1. *In vitro* digestion simulation.


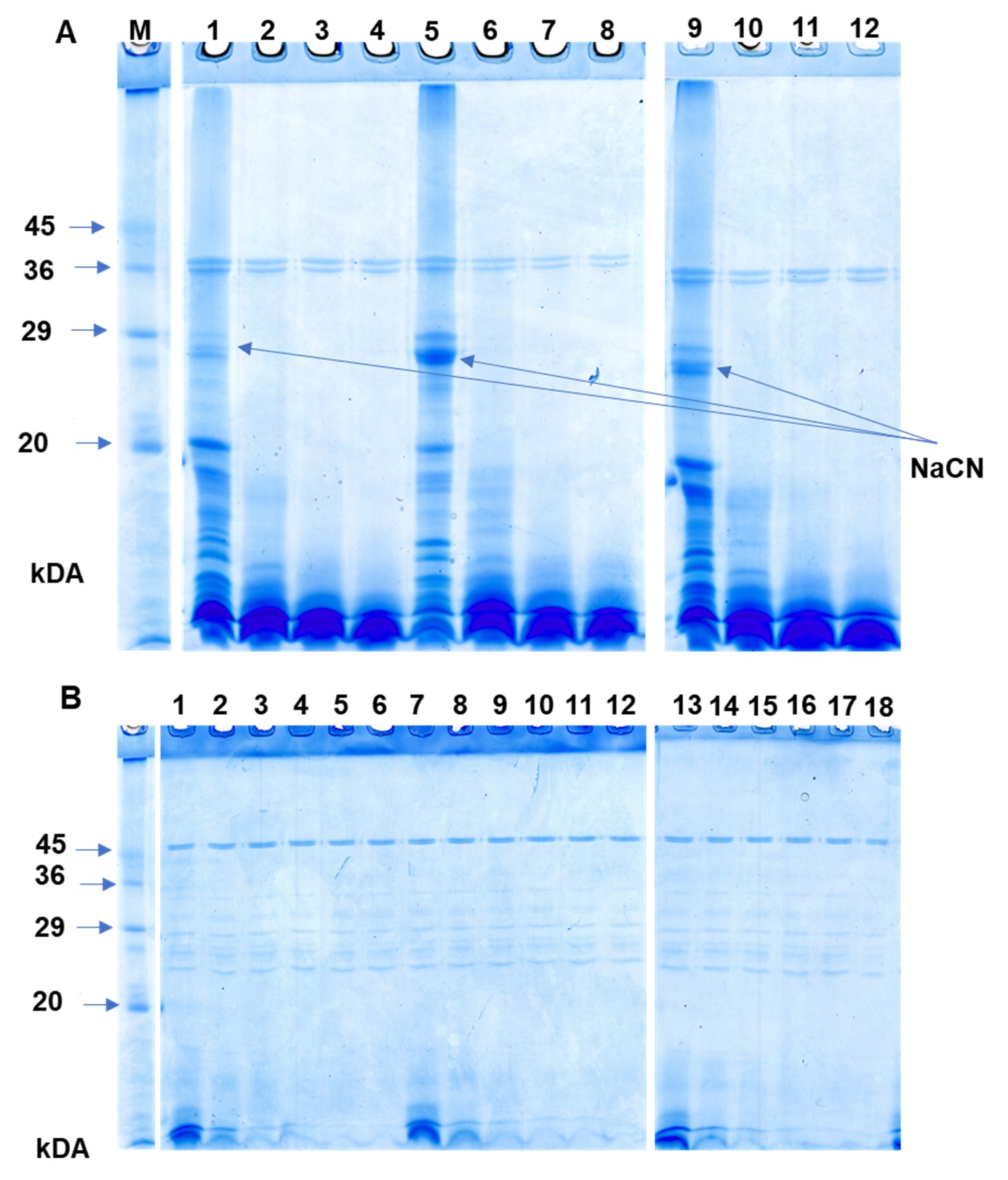


Figure S2. CN and CN interacted with SiO_2_ NPs during gastric (A) and intestinal (B) simulation. M: Marker; A1:NaCN G1; A2:NaCN G10; A3: NaCN G60; A4: NaCN G120; A5: NaCNHSi G1; A6: NaCNHSi G10; A7: NaCNHSi G60; A8: NaCNHSi G120; A9: NaCNLSi G1; A10: NaCNLSi G10; A11: NaCNLSi G60; A12: NaCNLSi G120; B1: NaCN I1; B2: NaCN I5; B3: NaCN I15; B4: NaCN I30; B5: NaCN I60; B6: NaCN I120; B7: NaCNHSi I1; B8: NaCNHSi I5; B9: NaCNHSi I15; B10: NaCNHSi I30; B11: NaCNHSi I60; B12: NaCNHSi I120; B13: NaCNLSi I1; B14: NaCNLSi I5; B15: NaCNLSi I15; B16: NaCNLSi I30; B17: NaCNLSi I60; B18: NaCNLSi I120


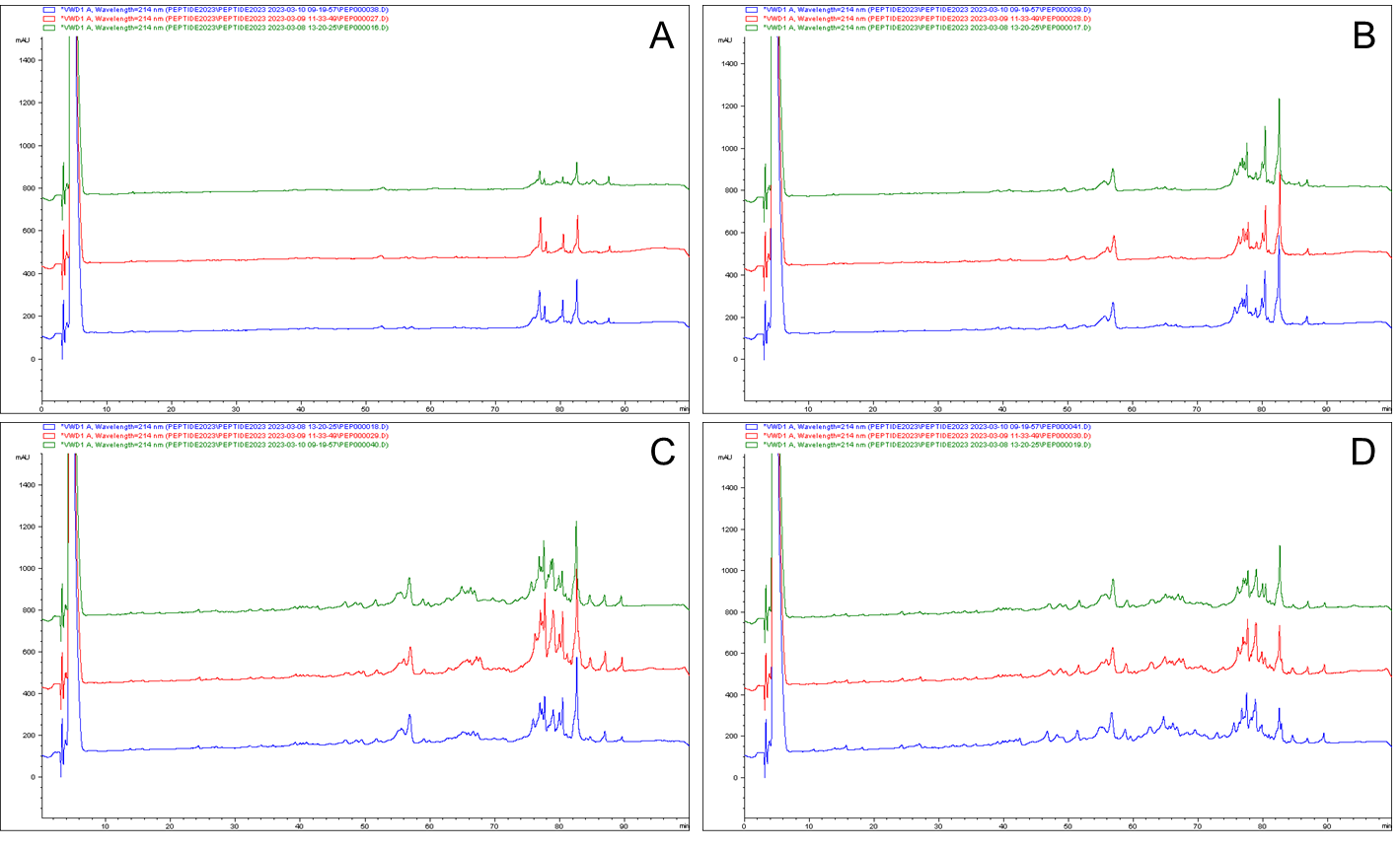


Fig. S3. Chromatograms of the samples with and without SiO_2_ NPs (NaCN (Blue), NaCNLSi (Red), NaCNHSi (Green)) at different time intervals during gastric digestion (A: 1 min, B: 30 min, C: 60 min and D: 120 min).


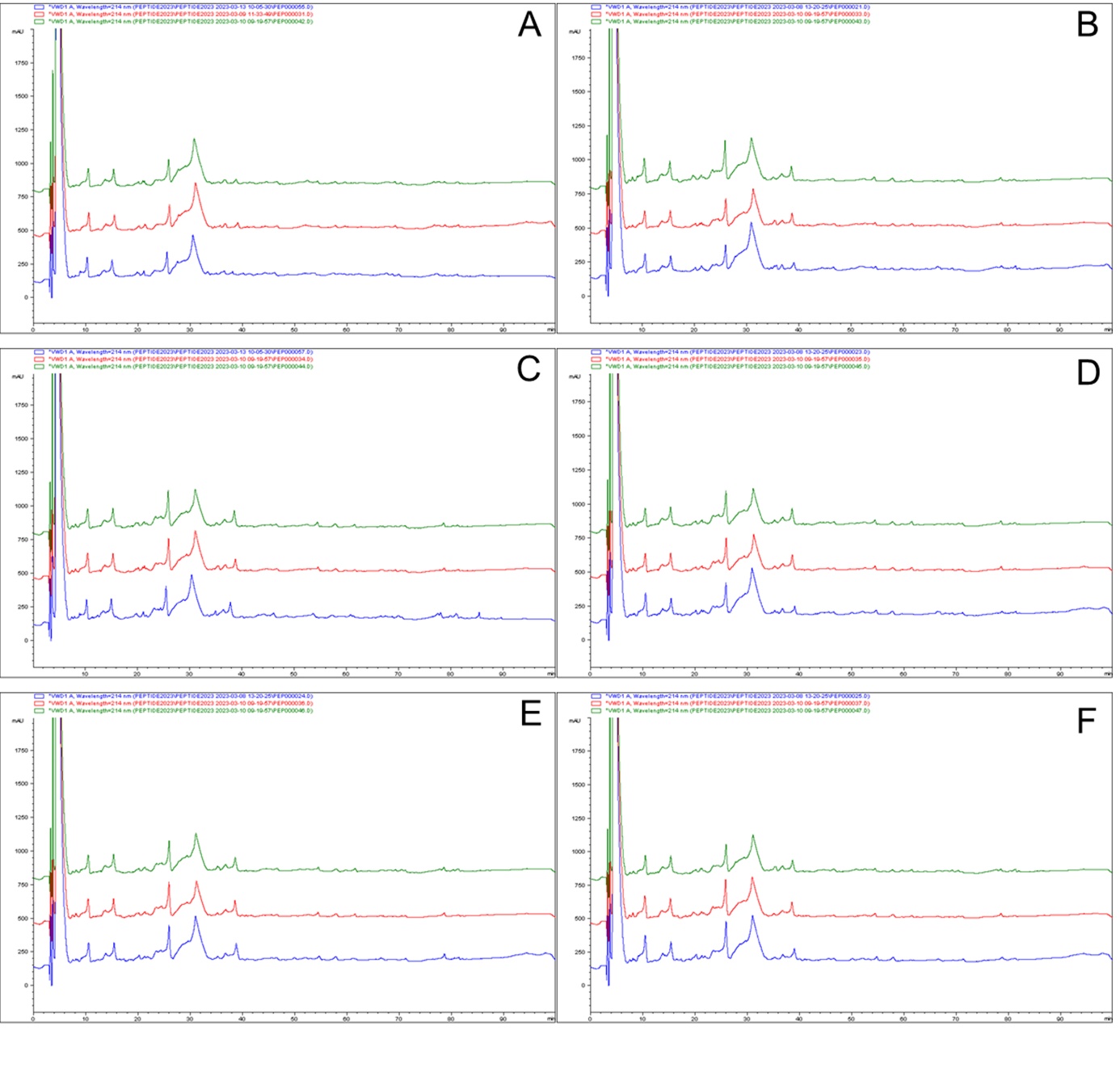


Fig. S4. Chromatograms of the samples with and without SiO_2_ NPs (NaCN (Blue), NaCNLSi (Red), NaCNHSi (Green)) at different time intervals during intestinal digestion (A: 1 min, B: 5 min, C: 15 min, D: 30 min, E: 60 min and F: 120 min).

Table S1. Inhibition/activation rate of the samples calculated in relation to the control (NaCN) at different time intervals representing the lag phase, logarithmic phase and stationary phase of microbial growth.

| Samples | % Inhibition/Activation | | | | | |
| --- | --- | --- | --- | --- | --- | --- |
|  | 60 min | | 190 min | | 360 min | |
|  | mean | std | mean | std | mean | std |
| NaCN | -5.58 | 5.52 | 5.42 | 2.42 | 1.69 | 1.77 |
| NaCNLSi | 5.06 | 3.97 | 3.54 | 3.30 | -0.16 | 1.86 |
| NaCNHSi | -5.06 | 14.39 | 2.31 | 2.53 | -0.84 | 1.08 |
